# Analysis of inequities between demographic and social determinants associated with overweight and obesity

**DOI:** 10.1590/1980-549720250038

**Published:** 2025-07-21

**Authors:** Pabyle Alves Flauzino, Ilana Nogueira Bezerra, Julia Sichieri Moura, Rosely Sichieri

**Affiliations:** I Universidade Estadual do Ceará, Postgraduate Program in Collective Health - Fortaleza (CE), Brazil.; II Universidade Estadual do Ceará, Postgraduate Program in Nutrition and Health - Fortaleza (CE), Brazil.; III Universidade Federal do Paraná, Postgraduate Program in Philosophy - Curitiba (PR), Brazil.; IV Universidade do Estado do Rio de Janeiro, Institute of Social Medicine, Department of Epidemiology - Rio de Janeiro (RJ), Brazil.

**Keywords:** Obesity, Intersectional framework, Social conditions, Race factors

## Abstract

**Objective:**

To assess demographic and social determinants associated with the prevalence of overweight and obesity from a population survey.

**Methods:**

Cross-sectional study with 28,153 adults participating in the 2017-2018 Household Budget Survey. Self-reported weight and height were used to estimate the prevalence of overweight and obesity, in addition to information on sociodemographic characteristics such as age (continuous), sex (male/female), skin color or race (white, brown, and black), location of residence (urban or rural), and *per capita* family income (quartile). Linear regression models were used to assess changes in mean BMI according to age and income in each area (urban/rural), stratified by sex and race/skin color.

**Results:**

In men, overweight and obesity were more prevalent in urban than rural areas, but there was no such difference in women. Income was quadratically related to the mean BMI among women, while in men the prevalence increased with income. In women, higher income was associated with lower BMI, except for black women, who did not show variation with income and were the ones with the highest prevalence of obesity. Men in rural areas had a much lower BMI and, respectively, a lower prevalence of obesity.

**Conclusion:**

We found no difference between extreme groups of possible inequality with regard to overweight and obesity. However, the prevalence of grade I obesity in men was different according to the area, being higher in men living in urban areas; and the highest prevalence of obesity according to race/color occurred among black women in the highest income quartile.

## INTRODUCTION

The prevalence of overweight and obesity in Brazilian adults has been increasing since the 1970s^1^. It is estimated that 56.8% of Brazilian adults were overweight (i.e., overweight and obese) in 2023^2^. However, the factors that predispose to the prevalence of overweight and obesity are not equally distributed among populations, including in Brazil^3^. Although diet, level of physical activity, sleep patterns, and other individual behavioral aspects are fundamental to understanding the predisposition to overweight, demographic and social determinants appear to have a direct influence on these inequities^4^.

The World Health Organization (WHO) defines equity as the absence of avoidable or irremediable differences between population groups, when defined by social, economic, demographic, or geographic criteria^5^. From a public health perspective, it is inequality - and not just inequality - that represents a central problem to be addressed^6^. For example, short stature in a given group may be characterized by a genetic alteration; on the other hand, when growth restriction occurs due to difficulty in providing adequate nutrition, there is inequality. Inequalities do not address whether differences are unfair or not, but inequities *per se* reflect a failure in equity and social justice. Gender roles and racism, historically present in Brazilian society, still subject women and black and brown people to economic and educational disparities and disparities in access to resources, which can harm their health and contribute to weight gain^7^ ^,^ ^8^.

In the national context, research focused on obesity does not address inequality, as it may be insufficient to understand the dynamics of health inequity. Demographic and social determinants, such as sex, race/skin color, area of ​​residence (i.e., urban or rural) and income can act as unfair and disproportionate barriers, making some groups marginalized and at greater risk of developing excess weight^9^. Considering that sex, race/skin color, area of ​​residence and income greatly influence the social conditions of individuals, the hypothesis of this study is that excess weight could be more prevalent among black women living in rural areas with the lowest income level, when compared to the reference group (i.e., white men living in urban areas with the highest income level) in the Brazilian population. Thus, in the present study, we evaluated demographic and social determinants associated with the prevalence of overweight and obesity from a population survey.

## METHODS

For the analyses in this article, data from the Household Budget Survey (POF), conducted in 2017-2018 by the Brazilian Institute of Geography and Statistics (IBGE) were used. POF is a household survey representative of the Brazilian population, with the purpose of investigating consumption strata, expenses, income and wealth variation of different population groups in all regions of the country. The data related to the 2017-2018 POF, which belongs to the public domain, can be obtained at: https://www.ibge.gov.br/estatisticas/sociais/saude/24786-pesquisa-deorcamentos-familiares-2.html?edicao=28523.

The sampling plan was conducted by clusters in two stages, involving the drawing of census sectors in the first stage and households in the second, based on a master sample defined in an Integrated Household Survey System (SIPD) of the IBGE, which employs a universal sample design in all household surveys. The Primary Sampling Units (UPAs) were composed of census sectors selected based on the census sectors of the 2010 Demographic Census. The UPAs were stratified both geographically and statistically, considering the geographic division (capitals, metropolitan regions, municipalities and urban or rural areas) and different socioeconomic levels. The selection of the UPAs was carried out by probabilistic sampling, according to the number of households in each stratum present in the master sample, and the households were selected by simple random sampling.

Sociodemographic information was collected from all residents of the selected households, and a subsample of residents over 10 years of age (34% of the households participating in the POF) provided food consumption data and information on weight and height. For this article, we used data from 28,153 adults (20 to 59 years old), excluding pregnant and nursing women, who responded to the food consumption survey with information on weight and height. Adolescents and older individuals were not included in the analyses, since other age-related factors (e.g., sexual maturation in adolescents and health conditions in older adults) may confound the relationship between demographic and social determinants and the prevalence of overweight and obesity.

Data were collected in households between July 2017 and July 2018. For these analyses, we used general information regarding sociodemographic characteristics (age, sex, skin color or race, and location of household - urban or rural area) and economic characteristics (monthly *per capita* household income), in addition to self-reported weight and height.

The prevalence rates and their respective 95% confidence intervals (95%CI) of overweight and obesity, stratified by sex, were estimated according to area (urban and rural), skin color (white, black, and brown), and income. *Per capita* family income was categorized into quartiles: 1st quartile up to R$649.20, 2nd quartile from R$649.20 to R$1,172.90, 3rd quartile from R$1,172.90 to R$2,069.40, and 4th quartile R$2,068.40 or more.

To assess changes in mean BMI according to age and income in each area (urban/rural), linear regression models were developed, with BMI as the dependent variable and age, income, and area as independent variables. To assess the linearity of the models, quadratic models were tested for age and income. To verify differences between groups, confidence intervals were used as reference. For income, the quadratic term was only statistically significant (p<0.05) for females. Effect modification by age was also assessed in the relationship between income quartiles and BMI variation, including age squared and multiplication of quadratic terms with income quartiles in the models, which did not obtain statistical significance. Linear regression models stratified by race were also developed. All analyses were stratified by sex, considering the complexity of the design and the sample weight, using the SAS (Statistical Analytics System) software, online version.

### Data Availability Statement

Data are public available at www.ibge.gov.br.

## RESULTS

The prevalence of overweight in the studied population was 38.7% (95%CI 37.8-39.6), being higher in men than in women (42.2%; 95%CI 40.9-43.5 vs. 35.8%; 95%CI 33.9-36.4, respectively). Obesity affected 16.7% (95%CI 15.8-17.7) of the individuals, being lower in men than in women (6.7%; 95%CI 5.6-7.7 vs. 11.3%; 95%CI 9.7-12.9, respectively). The prevalence of overweight and obesity in men differed according to area, being higher in men living in urban areas compared to those in rural areas, while in women there was no difference in prevalence between the areas. Regarding income, different behaviors were also observed between the sexes. While among men the prevalence increased with income, among women there was no difference in the prevalence of overweight and obesity between income groups. There was no difference in prevalence between race/skin color groups (Table 1). These results relate to the single-axis analyses of the characteristics of area, skin color, income and sex.

**Figure 1.**
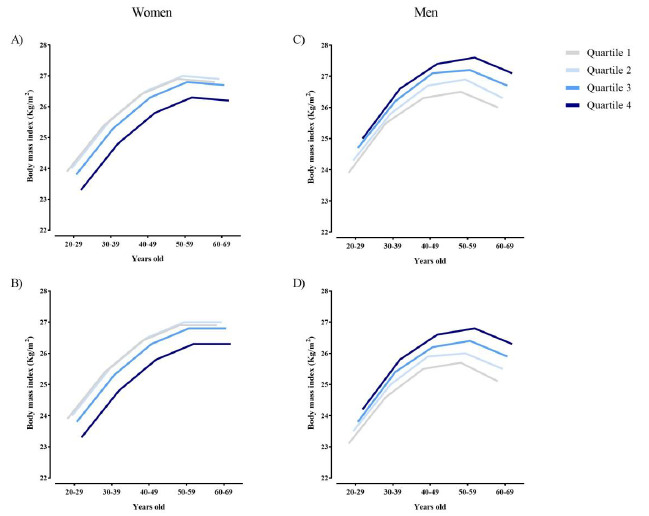
Average Body Mass Index (BMI) by age group and income quartiles, according to area (urban/rural) and sex. Household Budget Survey, 2017-2018. (A) Women Urban area. (B) Women Rural area. (C) Men Urban area. (D) Men Rural area.

Considering income, it was observed that there was a quadratic behavior in the average BMI among women, with the highest averages among women with lower incomes. These characteristics were observed in both urban and rural areas (Figure 1). Among men, the average BMI increased with increasing income level.

**Table 1.** Prevalence and 95% confidence interval (95%CI) of overweight and obesity, according to sociodemographic characteristics. Household Budget Survey, 2017-2018*.

	Men			Women		
	Overweight	Obesity 1	Obesity 2 and 3	Overweight	Obesity 1	Obesity 2 and 3
Area						
Urban	43.2 (41.7-44.7)	13.4 (12.4-14.5)	3.7 (3.2-4.3)	35.2 (33.8-36.6)	12.4 (11.5-13.3)	4.5 (4.0-5.1)
Rural	36.7 (34.6-38.9)	9.6 (8.1-11.0)	1.7 (1.1-2.4)	35.1 (32.4-37.8)	14.1 (12.3-15.8)	4.8 (3.8-5.8)
Income						
Quartile 1	38.3 (36.0-40.7)	10.3 (8.8-11.7)	2.0 (1.4-2.6)	34.9 (32.7-37.1)	13.2 (11.7-14.7)	5.4 (4.4-6.3)
Quartile 2	40.5 (38.0-43.0)	11.8 (10.3-13.3)	3.1 (2.3-4.0)	36.0 (33.6-38.3)	12.5 (11.0-13.9)	4.7 (3.8-5.6)
Quartile 3	44.0 (41.3-46.7)	13.8 (12.0-15.6)	3.5 (2.5-4.4)	36.2 (33.7-38.7)	14.0 (12.2-15.9)	4.4 (3.4-5.4)
Quartile 4	45.6 (42.6-48.6)	15.4 (13.2-17.5)	5.0 (3.7-6.3)	33.6 (30.9-36.4)	10.7 (8.9-12.6)	3.8 (2.7-4.8)
Race						
While	42.8 (40.8-44.9)	13.0 (11.5-14.4)	4.1 (3.3-5.0)	35.7 (33.7-37.7)	11.4 (10.1-12.7)	4.2 (3.5-4.9)
Brown	42.4 (40.4-44.4)	12.7 (11.4-14.0)	2.7 (2.2-3.3)	34.2 (32.5-36.0)	13.4 (12.2-14.6)	4.9 (4.2-5.6)
Black	38.7 (34.7-42.7)	12.8 (10.3-15.4)	3.8 (2.0-5.6)	38.8 (34.8-42.9)	14.2 (11.7-16.7)	5.2 (3.7-6.8)

*Results presented in proportion (%) and confidence interval (CI).

Among men of white and brown race/skin color, it was observed that the prevalence of obesity increased with increasing income quartile, although this behavior did not occur among black men. Higher prevalences of obesity were observed in all categories of race/skin color among men in the highest income quartile. Among women of white race/skin color, the prevalence of obesity decreases with increasing income quartile. Brown and black women do not show a dose-response relationship in the relationship between BMI and income. Higher prevalence of obesity is observed in brown women in the third income quartile and in black women in the fourth income quartile. These results are shown in Table 2.

**Table 2.** Prevalence and 95% confidence interval (95%CI) of obesity between race/color, sex and income quartile. Household Budget Survey, 2017-2018*.

	Income			
	Quartile 1	Quartile 2	Quartile 3	Quartile 4
Men				
White	9.7 (6.9-12.4)	15.8 (12.8-18.9)	18.5 (15.5-21.5)	19.9 (16.8-22.9)
Brown	12.5 (10.5-14.4)	14.4 (12.1-16.6)	16.8 (13.6-20.1)	20.3 (15.9-24.6)
Black	16.7 (11.2-22.1)	15.5 (10.7-20.3)	14.3 (9.8-18.8)	21.7 (12.9-30.5)
Women				
While	19.4 (15.4-23.4)	16.4 (13.4-19.4)	15.9 (13.1-18.7)	13.3 (10.7-15.9)
Brown	18.8 (16.7-21.0)	16.4 (14.4-18.3)	21.4 (17.9-24.9)	15.5 (11.6-19.3)
Black	16.0 (11.5-20.6)	23.4 (17.2-29.6)	16.9 (11.2-22.7)	24.4 (16.2-32.6)

*Results presented in proportion (%) and confidence interval (CI).

Table 3 presents the results of the magnitudes of the associations between income and area, with the increase in BMI stratified by sex and race/skin color. Women of white race/skin color remain statistically associated with a reduction in BMI with each increase in income quartile, showing an inverse relationship. Income did not influence BMI in brown and black women (p>0.05). The region of residence (rural/urban) also does not appear to influence the increase in BMI in any category of race/skin color in women (p>0.05). In men of white and brown race/skin color, the increase in the income quartile was statistically associated with the increase in BMI. In black men, income and living in rural areas also did not seem to influence the variation in BMI (p>0.05), although living in rural areas was associated with lower BMI values ​​among white and brown men.

**Table 3.** Regression coefficients* and 95% confidence interval (95%CI) of body mass index (BMI=kg/m^2^) according to sex, skin color, income in quartiles and area (rural/urban). Household Budget Survey, 2017-2018.

	Women			Men		
	While	Brown	Black	While	Brown	Black
n	5,500	7,685	1,456	4,851	6,847	1,507
Income 95%CI	-0.28 (-0.47 a -0.09)	-0.06 (-0.22-0.10)	0.02 (-0.30-0.35)	0.37 (0.21-0.54)	0.42 (0.27-0.58)	0.14 (-0.19-0.46)
p value	0.0045	0.4638	0.8817	<0.001	<0.001	0.4091
Area 95%CI	0.17 (-0.30-0.64)	-0.07 (-0.47-0.32)	0.17 (-0.96-1.30)	-0.81(-1.17 a -0.45)	-0.81 (-1.14 a -0.48)	-0.79 (-1.80-0.22)
p value	0.4949	0.7044	0.7684	<0.001	<0.001	0.1266

*mutually adjusted model for age and age*age

## DISCUSSION

In this study, we sought to compare the extremes of possible inequality in terms of obesity between two groups: black women living in rural areas with lower income levels versus white men living in urban areas with higher income levels. Furthermore, we found that the prevalence of grade I obesity in men differed according to the area, being higher in men living in urban areas, and the highest prevalence of obesity according to race/skin color occurred among black women in the highest income quartile when compared to white women in the highest income quartile. In both black men and women, there was no association between income and BMI.

We found no differences between black women living in rural areas with lower income levels versus white men living in urban areas with higher income levels. This fact can possibly be explained by the complexity of the issues related to the race/skin color variable, since the data from the household budget survey may not be sufficient to reflect the social dynamics related to this variable^8^. First, the official classification of race in Brazil is based on self-identification in five categories^10^. The category “brown” is a descriptor for black or indigenous people with white ancestry^10^. As a result, the category “brown” is quite flexible, giving individuals an ambiguous phenotype (i.e., they can be considered white or black simultaneously, according to them or others)^11^. However, for the Brazilian reality, this cannot be the only hypothesis, given that this argument can limit/weaken the discussion of racial inequities. Another hypothesis for this is due to hormonal and reproductive health differences - such as pregnancy and menopause -, macrostructural conditions - such as the use of agricultural pesticides^12^ and other substances considered endocrine disruptors -, which can explain the disparities in composition between the sexes, although they are not sufficient. The specific experiences of men and women in relation to obesity can be shaped by specific factors, such as those resulting from a racist patriarchal structure^13^ ^,^ ^14^. For example, social expectations surrounding the female and male body, as well as gender- and race-specific cultural norms, play a crucial role in shaping attitudes toward eating, body image, and exercise practices^15^.

Comparisons with other studies should be cautious, given that despite some similarities in race/skin color classifications, being black, white, Asian/yellow, etc., there are large discrepancies in different historical and social contexts. A recent review pointed out that most studies on obesity come from the American population and stated that the growing obesity epidemic is unequal between blacks and whites, even after adjusting for age and socioeconomic status, reiterating the specificity of racial inequalities beyond social class differences^16^.

In this study, we found that the prevalence of grade 1 obesity was higher among men in urban areas. These findings are consistent with the literature, since these areas differ according to their demographic, cultural and socioeconomic characteristics, which determine the health of the subjects in different ways^17^ ^,^ ^18^. A possible explanation for this finding is the greater availability of ultra-processed foods, pollution, sleep deprivation and stress in urban areas, with all of these factors strongly associated with excess body fat accumulation^19^ ^,^ ^20^.

In our findings, black women in the highest income quartile had a higher prevalence of obesity when compared to white women in the highest income quartile. This finding adds to a paradoxical body of evidence demonstrating a higher prevalence of obesity among socially advantaged populations in low- and middle-income countries^21^ ^,^ ^22^. For black women with high income and education, the high prevalence of overweight may be justified by a “hidden” form of inequality, originating from a racist structure, in which discrimination can generate negative consequences for body weight and health, unlike their peers^23^. Another hypothesis for the aforementioned fact is the possibility that black women have greater body mass and are less susceptible to the white Western ideal of thinness. Thus, black women may be less likely to engage in body modification activities that culminate in weight loss^24^.

The obesity epidemic is multifactorial and its determinants are complex^25^ ^,^ ^26^. Several biological factors have been investigated, and many play a role in the accumulation of body fat, but there is no single theory that can truly explain the epidemic. A consensus has been prevailing, which concerns the concept of metabolic balance. However, population data (2000-2018) refute the positive energy balance hypothesis, showing an increase in obesity from 31 to 42%, with no evidence of increased energy intake or decreased physical activity^27^ ^,^ ^28^.

Among the various biological factors and theories about fat accumulation, there are exposures to more than 50 obesogens - chemicals that alter endocrine signaling, known as disruptors, or that alter the body’s functions, including insulin levels and metabolic rate - whose changes can even be transmitted through generations^29^. There is also ample documentation that excessive consumption of sugars and ultra-processed products leads to excessive weight gain^28^. On the other hand, new medications that reduce appetite are promising in terms of weight loss and weight maintenance^30^.

Policies, programs, and guidelines for the prevention and control of obesity in Brazil, even though they consider its multifactorial nature, have an excessive focus on the biological aspect^31^. This fact ignores the intersections of gender/sex, race/color, geographic space, and social class, which greatly contribute to the predisposition to or worsening of obesity. This study sheds light on these categories that have been little analyzed with regard to obesity. Thus, we indicate which factors may be most related to weight gain, which can help guide obesity prevention and control policies, such as the National Food and Nutrition Policy^32^. Thus, nutritional surveillance strategies, monitoring of the risk of food insecurity and the promotion of adequate and healthy eating become more targeted at disadvantaged groups and can make the National Food and Nutrition Policy more effective and fair.

Despite the important findings, the limitations of this study include:

• the complexity of self-declaration of skin color/race, which may not adequately capture the racial phenomenon;
• the data are based on the household budget survey, which has as its main objective to investigate income and expenses among Brazilian families, and does not have specific variables focusing on health inequities;
• the information on weight and height was self-declared, so memory bias may compromise the accuracy of the data.

Although weight and height are self-reported, both can be used to calculate BMI and classify nutritional status in a valid and rapid manner in population surveys^33^ ^,^ ^34^. As strengths, we highlight the population representativeness of the Household Budget Survey with demographic and socioeconomic data that allow for the assessment of the population’s health conditions. As observed in the present study, although higher income is historically associated with increased opportunity for resources, high income can influence health and nutrition conditions differently depending on race and gender. Thus, the present study adds to the field of obesity epidemiology by shedding light on inequities, especially according to race/color, sex and income.

In conclusion, we found no differences between groups located at the extremes of possible inequity with regard to obesity. However, the prevalence of grade I obesity in men was different according to the area, being higher in men living in urban areas. A higher prevalence of obesity was also observed according to race/color, among black women in the highest income quartile. However, we found differences in the social pattern of prevalence of overweight and obesity in the population with regard to sex, race/color and income.
