# Supplementary material for: Analysis of inequities between demographic and social determinants associated with overweight and obesity
Source: Rev Bras Epidemiol. 2025 Jul 21;28:e250038. doi: 10.1590/1980-549720250038 (PMC12278848; doi:10.1590/1980-549720250038)
Supplement: Translation

# Translation: Análise de iniquidades entre determinantes demográficos e sociais associados ao sobrepeso e obesidade

**DOI:** 10.1590/1980-549720250038.2

**Authors:** Pabyle Alves Flauzino, Ilana Nogueira Bezerra, Julia Sichieri Moura, Rosely Sichieri

**Affiliations:** I Universidade Estadual do Ceará, Programa de Pós-graduação em Saúde Coletiva - Fortaleza (CE), Brasil.; II Universidade Estadual do Ceará, Programa de Pós-graduação em Nutrição e Saúde - Fortaleza (CE), Brasil.; III Universidade Federal do Paraná, Programa de Pós-Graduação em Filosofia - Curitiba (PR), Brasil.; IV Universidade do Estado do Rio de Janeiro, Instituto de Medicina Social, Departamento de Epidemiologia - Rio de Janeiro (RJ), Brasil.

**Keywords:** Obesidade, Enquadramento interseccional, Condições sociais, Fatores raciais

## Abstract

**Objetivo:**

Avaliar determinantes demográficos e sociais associados à prevalência de sobrepeso e obesidade provenientes de inquérito populacional.

**Métodos:**

Estudo transversal com 28.153 adultos participantes da Pesquisa de Orçamento Familiar 2017-2018. Utilizou-se peso e altura autorreferidos para estimar a prevalência de sobrepeso e obesidade, além de informações sobre características sociodemográficas, como idade (contínua), sexo (homem/mulher), cor ou raça (branca, parda e preta), localização do domicílio (urbano ou rural), e renda familiar per capita (quartil). Modelos de regressão linear foram usados para avaliar as mudanças na média de IMC segundo idade e renda em cada área (urbano/rural), com estratificação por sexo e raça/cor da pele.

**Resultados:**

Nos homens, o sobrepeso e a obesidade foram mais prevalentes em áreas urbanas do que rurais, mas nas mulheres não houve essa diferença. A renda relaciona-se de forma quadrática com a média do IMC entre as mulheres, enquanto nos homens a prevalência aumenta com a renda. Nas mulheres, maiores rendas associam-se a menor IMC, exceto para as pretas que não apresentam variação com a renda e são as que apresentam as maiores prevalências de obesidade. Já os homens na zona rural têm IMC muito menor e respectivamente menor prevalência de obesidade.

**Conclusão:**

Não encontramos diferenças entre os grupos situados em extremos de possível iniquidade no que diz respeito ao sobrepeso e obesidade. Entretanto, a prevalência de obesidade grau I nos homens foi diferente de acordo com a área, sendo maior nos homens residentes da área urbana; e a maior prevalência de obesidade de acordo com a raça/cor se deu entre as mulheres pretas no maior quartil de renda.

## INTRODUÇÃO

A prevalência de sobrepeso e obesidade em adultos brasileiros tem aumentado desde a década de 1970^1^. Estima-se que 56,8% dos adultos brasileiros possuíam excesso de peso (i.e., sobrepeso e obesidade) no ano de 2023^2^. No entanto, os fatores que predispõem a prevalência de sobrepeso e obesidade não estão distribuídos igualmente entre as populações, inclusive no Brasil^3^. Embora a dieta, o nível de atividade física, o padrão de sono e outros aspectos comportamentais individuais sejam fundamentais para a compreensão da predisposição do excesso de peso, determinantes demográficos e sociais parecem exercer influência direta para essas iniquidades^4^.

A Organização Mundial da Saúde (OMS) define equidade como a ausência de diferenças evitáveis ou irremediáveis entre grupos da população, quando são definidos por critérios sociais, econômicos, demográficos ou geográficos^5^. Na perspectiva da Saúde Pública, é a iniquidade - e não apenas a desigualdade - que representa um problema central a ser enfrentado^6^. Por exemplo, a baixa estatura em determinado grupo pode caracterizar uma alteração genética; por outro lado, quando a restrição de crescimento ocorre por dificuldade para prover alimentação adequada, há iniquidade^6^. As desigualdades não abordam se as diferenças são injustas ou não, mas as iniquidades *per si* refletem a falha na equidade e na justiça social. Os papéis de gênero e o racismo, historicamente presentes na sociedade brasileira ainda submetem mulheres e pessoas pretas e pardas a disparidades econômicas, educacionais e no acesso a recursos, o que pode prejudicar sua saúde e contribuir para o ganho de peso^7^ ^,^ ^8^.

No contexto nacional, as investigações voltadas para a obesidade devem superar apenas a desigualdade, pois podem ser insuficientes por não compreenderem a dinâmica de iniquidades em saúde. Determinantes demográficos e sociais, como o sexo, raça/cor, área de moradia (i.e., urbano ou rural) e renda podem atuar como barreiras injustas e desproporcionais, tornando alguns grupos marginalizados e com maior risco de desenvolver excesso de peso^9^. Considerando que sexo, raça/cor da pele, área de moradia e renda influenciam sobremaneira as condições sociais dos sujeitos, a hipótese deste estudo é que o excesso de peso poderia ter maior prevalência entre mulheres pretas vivendo em área rural no menor nível de renda, quando comparadas ao grupo de referência (i.e., homens brancos vivendo em área urbana no maior nível de renda) na população brasileira. Desta maneira, no presente estudo, avaliamos determinantes demográficos e sociais associados à prevalência de sobrepeso e obesidade provenientes de inquérito populacional.

## MÉTODOS

Para as análises deste artigo, utilizou-se os dados da Pesquisa de Orçamento Familiar (POF), realizada em 2017-2018 pelo Instituto Brasileiro de Geografia e Estatística (IBGE). A POF é um inquérito domiciliar representativo da população brasileira, com a finalidade de investigar estratos de consumo, despesas, rendimentos e variação patrimonial dos diferentes grupos populacionais em todas as regiões do país. A leitura dos dados referentes à POF 2017-2018, pertencentes ao domínio público, pode ser realizada em: https://www.ibge.gov.br/estatisticas/sociais/saude/24786-pesquisa-deorcamentos-familiares-2.html?edicao=28523.

O plano de amostragem foi conduzido por conglomerados em dois estágios, envolvendo o sorteio de setores censitários no primeiro estágio, e de domicílios no segundo, baseados em uma amostra-mestra definida em um Sistema Integrado de Pesquisas Domiciliares (SIPD) do IBGE, que emprega um desenho de amostra universal em todas as pesquisas domiciliares. As Unidades Primárias de Amostragem (UPAs) foram compostas por setores censitários selecionados com base nos setores censitários do Censo Demográfico de 2010. As UPAs foram estratificadas tanto geograficamente quanto estatisticamente, considerando a divisão geográfica (capitais, regiões metropolitanas, municípios e áreas urbanas ou rurais) e diferentes níveis socioeconômicos. A seleção das UPAs foi realizada por amostragem probabilística, de acordo com o número de domicílios em cada estrato presente na amostra-mestra, e os domicílios foram selecionados por amostragem aleatória simples.

Informações sociodemográficas foram perguntadas para todos os moradores dos domicílios selecionados e uma subamostra de moradores acima de 10 anos de idade de 34% dos domicílios participantes da POF forneceram dados de consumo alimentar e informação sobre peso e altura. Para este artigo, utilizamos os dados de 28.153 indivíduos adultos (20 a 59 anos), excluindo-se grávidas e nutrizes, que responderam ao inquérito de consumo alimentar com informação sobre peso e altura. Adolescentes e idosos não foram incluídos nas análises, pois outros fatores inerentes à idade (por exemplo, maturação sexual nos adolescentes e condições de saúde em idosos) podem confundir a relação entre determinantes demográficos e sociais e prevalência de sobrepeso e obesidade.

Os dados foram coletados nos próprios domicílios, entre Julho de 2017 e Julho de 2018. Para estas análises, utilizamos informações gerais relativas às características sociodemográficas (idade, sexo, cor ou raça e localização do domicílio - área urbana ou rural) e econômicas (renda familiar per capita mensal), além do peso e altura autorreferidos.

As prevalências e seus respectivos intervalos de confiança 95% (IC95%) de sobrepeso e obesidade, estratificados por sexo, foram estimados segundo a área (urbana e rural), cor da pele (branca, preta e parda) e renda. A renda familiar *per capita* foi categorizada em quartis: 1° quartil até R$ 649,20, 2° quartil de R$ 649,20 a R$ 1.172,90, 3° quartil de R$ 1.172,90 a R$ 2.069,40 e 4° quartil R$ 2.068,40 ou mais.

Para avaliar as mudanças na média de IMC segundo idade e renda em cada área (urbano/rural), modelos de regressão linear foram desenvolvidos, com IMC como variável dependente, e idade, renda e área como variáveis independentes. Para avaliar a linearidade dos modelos, foram testados modelos quadráticos para idade e renda. Para verificar as diferenças entre os grupos, utilizou-se como referência os intervalos de confiança. Para renda, o termo quadrático só foi estatisticamente significante (p<0,05) para o sexo feminino. Avaliou-se também a modificação de efeito pela idade, na relação entre quartis de renda e variação do IMC, incluído nos modelos a idade ao quadrado e a multiplicação dos termos quadráticos com os quartis de renda, que não obtiveram significância estatística. Modelos de regressão linear estratificados por raça também foram desenvolvidos. Todas as análises foram estratificadas por sexo, considerando a complexidade do desenho e o peso amostral, usando o software *SAS* (*Statistical Analytics System*) versão online.

### Declaração de disponibilidade de dados

Os dados são públicos e estão disponíveis em: www.ibge.gov.br.

## RESULTADOS

A prevalência de sobrepeso na população investigada foi de 38,7% (Intervalo de confiança de 95% - IC95% 37,8-39,6), sendo maior nos homens do que nas mulheres (42,2%; IC95% 40,9-43,5 vs. 35,8%; IC95% 33,9-36,4, respectivamente). Já a obesidade acometeu 16,7% (IC95% 15,8-17,7) dos indivíduos, sendo menor nos homens do que nas mulheres (6,7%; IC95% 5,6-7,7 vs. 11,3%; IC95% 9,7-12,9, respectivamente). A prevalência de sobrepeso e obesidade nos homens foi diferente de acordo com a área, sendo maior nos homens residentes de área urbana em comparação com os de área rural, enquanto nas mulheres não houve diferença nas prevalências entre as áreas. Quanto à renda, também se observa comportamentos distintos entre os sexos. Enquanto nos homens a prevalência aumenta com a renda, entre as mulheres não há diferença da prevalência de sobrepeso e obesidade entre as faixas de renda. Não houve diferença nas prevalências entre as raças/cor da pele (Tabela 1). Esses resultados dizem respeito às análises de eixo único das características de área, cor da pele, renda e sexo.

**Tabela 1.** Prevalência e intervalo de confiança de 95% (IC95%) de sobrepeso e obesidade, segundo características sociodemográficas. Pesquisa de Orçamento Familiar, 2017-2018*.

	Homem			Mulher		
	Sobrepeso	Obesidade 1	Obesidade 2 e 3	Sobrepeso	Obesidade 1	Obesidade 2 e 3
Área						
Urbano	43,2 (41,7-44,7)	13,4 (12,4-14,5)	3,7 (3,2-4,3)	35,2 (33,8-36,6)	12,4 (11,5-13,3)	4,5 (4,0-5,1)
Rural	36,7 (34,6-38,9)	9,6 (8,1-11,0)	1,7 (1,1-2,4)	35,1 (32,4-37,8)	14,1 (12,3-15,8)	4,8 (3,8-5,8)
Renda						
Quartil 1	38,3 (36,0-40,7)	10,3 (8,8-11,7)	2,0 (1,4-2,6)	34,9 (32,7-37,1)	13,2 (11,7-14,7)	5,4 (4,4-6,3)
Quartil 2	40,5 (38,0-43,0)	11,8 (10,3-13,3)	3,1 (2,3-4,0)	36,0 (33,6-38,3)	12,5 (11,0-13,9)	4,7 (3,8-5,6)
Quartil 3	44,0 (41,3-46,7)	13,8 (12,0-15,6)	3,5 (2,5-4,4)	36,2 (33,7-38,7)	14,0 (12,2-15,9)	4,4 (3,4-5,4)
Quartil 4	45,6 (42,6-48,6)	15,4 (13,2-17,5)	5,0 (3,7-6,3)	33,6 (30,9-36,4)	10,7 (8,9-12,6)	3,8 (2,7-4,8)
Raça						
Branca	42,8 (40,8-44,9)	13,0 (11,5-14,4)	4,1 (3,3-5,0)	35,7 (33,7-37,7)	11,4 (10,1-12,7)	4,2 (3,5-4,9)
Parda	42,4 (40,4-44,4)	12,7 (11,4-14,0)	2,7 (2,2-3,3)	34,2 (32,5-36,0)	13,4 (12,2-14,6)	4,9 (4,2-5,6)
Preta	38,7 (34,7-42,7)	12,8 (10,3-15,4)	3,8 (2,0-5,6)	38,8 (34,8-42,9)	14,2 (11,7-16,7)	5,2 (3,7-6,8)

*Resultados apresentados em proporção (%) e Intervalo de confiança (IC).

Considerando a renda, observa-se que há um comportamento quadrático na média do IMC entre as mulheres, sendo as maiores médias entre as mulheres de menores rendas. Essas características são observadas tanto na área urbana quanto na rural (Figura 1). Já entre os homens, a média do IMC aumenta com o aumento do nível de renda.

**Figura 1.** Média do Índice de Massa Corporal (IMC) por faixa etária e quartis de renda, segundo área (urbana/rural) e sexo. Pesquisa de Orçamento Familiar, 2017-2018. (A) Mulher Urbana. (B) Mulher área Rural. (C) Homem área Urbana. (D) Homem área Rural.

Entre os homens de raça/cor da pele branca e parda, observou-se que a prevalência de obesidade aumenta com o incremento do quartil de renda, sendo que esse comportamento não acontece nos homens pretos. Maiores prevalências de obesidade são observadas em todas as categorias de raça/cor da pele nos homens do maior quartil de renda. Já entre as mulheres de raça/cor da pele branca a prevalência de obesidade diminui com o incremento do quartil de renda. Mulheres pardas e pretas não apresentam uma relação de dose-resposta na relação entre IMC e renda. Maiores prevalências de obesidade são observadas nas mulheres pardas no terceiro quartil de renda e nas mulheres pretas no quarto quartil de renda. Esses resultados estão expressos na Tabela 2.

**Tabela 2.** Prevalência e intervalo de confiança de 95% (IC95%) de obesidade entre raça/cor, sexo e quartil de renda. Pesquisa de Orçamento Familiar, 2017-2018*.

	Renda			
	Quartil 1	Quartil 2	Quartil 3	Quartil 4
Homem				
Branca	9,7 (6,9-12,4)	15,8 (12,8-18,9)	18,5 (15,5-21,5)	19,9 (16,8-22,9)
Parda	12,5 (10,5-14,4)	14,4 (12,1-16,6)	16,8 (13,6-20,1)	20,3 (15,9-24,6)
Preta	16,7 (11,2-22,1)	15,5 (10,7-20,3)	14,3 (9,8-18,8)	21,7 (12,9-30,5)
Mulher				
Branca	19,4 (15,4-23,4)	16,4 (13,4-19,4)	15,9 (13,1-18,7)	13,3 (10,7-15,9)
Parda	18,8 (16,7-21,0)	16,4 (14,4-18,3)	21,4 (17,9-24,9)	15,5 (11,6-19,3)
Preta	16,0 (11,5-20,6)	23,4 (17,2-29,6)	16,9 (11,2-22,7)	24,4 (16,2-32,6)

*Resultados apresentados em proporção (%) e Intervalo de confiança (IC).

A Tabela 3 apresenta os resultados das magnitudes das associações entre renda e área, com o aumento do IMC estratificado por sexo e raça/cor da pele. As mulheres de raça/cor da pele branca a cada aumento no quartil de renda permanecem associadas estatisticamente com uma redução no IMC, apresentando uma relação inversa. A renda não influenciou no IMC em mulheres pardas e pretas (p>0,05). A região de moradia (rural/urbano) também parece não influenciar no aumento do IMC em nenhuma categoria de raça/cor da pele em mulheres (p>0,05). Já em homens de raça/cor da pele branca e parda, o aumento no quartil de renda foi associado estatisticamente com o aumento no IMC. Em homens pretos, a renda e morar na zona rural também parecem não influenciar na variação do IMC (p>0,05), embora morar na zona rural foi associado a menores valores de IMC entre brancos e pardos.

**Tabela 3.** Coeficientes de regressão* e intervalo com 95% de confiança (IC95%) do índice de massa corporal (IMC=kg/m^2^) segundo sexo, cor de pele, renda em quartis e área (rural/urbano). Pesquisa de Orçamento Familiar, 2017-2018.

	Mulher			Homem		
	Branca	Parda	Preta	Branco	Pardo	Preto
n	5.500	7.685	1.456	4.851	6.847	1.507
Renda IC95%	-0,28 (-0,47 a -0,09)	-0,06 (-0,22-0,10)	0,02 (-0,30-0,35)	0,37 (0,21-0,54)	0,42 (0,27-0,58)	0,14 (-0,19-0,46)
Valor p	0,0045	0,4638	0,8817	<0,001	<0,001	0,4091
Área IC95%	0,17 (-0,30-0,64)	-0,07 (-0,47-0,32)	0,17 (-0,96-1,30)	-0,81 (-1,17 a -0,45)	-0,81 (-1,14 a -0,48)	-0,79 (-1,80-0,22)
Valor p	0,4949	0,7044	0,7684	<0,001	<0,001	0,1266

* modelo mutuamente ajustado por idade e idade*idade.

## DISCUSSÃO

Neste estudo, buscamos comparar os extremos de possível iniquidade, no que diz respeito à obesidade, entre dois grupos: mulher negra, vivendo na área rural e com menor nível de renda, *versus* homem branco, vivendo em área urbana e com maior nível de renda. Ainda, encontramos que a prevalência de obesidade grau I nos homens foi diferente de acordo com a área, sendo maior nos homens residentes da área urbana; e a maior prevalência de obesidade de acordo com a raça/cor se deu entre as mulheres pretas no maior quartil de renda quando comparadas a mulheres brancas no maior quartil de renda. Tanto nos homens quanto nas mulheres pretas não há associação da renda com o IMC.

Não encontramos diferenças entre mulheres pretas, vivendo na área rural e com menor nível de renda, *versus* homem branco, vivendo em área urbana e com maior nível de renda. Tal fato possivelmente pode ser explicado pela complexidade das questões da variável raça/cor da pele, uma vez que os dados da pesquisa de orçamento familiares podem não ser suficientes para refletir as dinâmicas sociais relacionadas a essa variável^8^. Primeiro, a classificação oficial de raça no Brasil se dá pela autoidentificação em cinco categorias^10^. A categoria “pardo” é um descritor para pessoas negras ou indígenas com ascendência branca^10^. Com isto, a categoria “pardo” é bastante elástica, conferindo aos indivíduos um fenótipo ambíguo (i.e., podem ser considerados brancos ou pretos em concomitância, por si ou por terceiros)^11^. No entanto, para a realidade brasileira essa não pode ser a única hipótese, tendo em vista que este argumento pode limitar/fragilizar a discussão das iniquidades raciais. Outra hipótese para tal se dá pelas diferenças hormonais e de saúde reprodutiva - como a gravidez e a menopausa -, condições macroestruturais - como o uso de pesticidas agrícolas^12^ e outras substâncias consideradas como disruptores endócrinos -, que podem explicar as disparidades de composição entre os sexos, ainda que não sejam suficientes. As experiências específicas de homens e mulheres em relação à obesidade podem ser moldadas por fatores específicos, como decorrentes de uma estrutura racista patriarcal^13^ ^,^ ^14^. Por exemplo, as expectativas sociais em torno do corpo feminino e masculino, bem como normas culturais específicas para cada sexo e raça, desempenham um papel crucial na formação das atitudes em relação à alimentação, imagem corporal e práticas de exercício^15^.

Comparações com outros estudos devem ser cautelosas, tendo em vista que, apesar de algumas similaridades nas classificações de raça/ cor da pele, ser negro, branco, asiático/amarelo etc., em contextos históricos e sociais diferentes, possui grandes discrepâncias. Uma revisão recente apontou que a maioria dos estudos sobre obesidade são provenientes da população americana e afirmou que a crescente epidemia de obesidade é desigual entre negros e brancos, mesmo após ajuste para idade e nível socioeconômico, reiterando a especificidade das desigualdades raciais para além das diferenças de classe social^16^.

Neste estudo, encontramos que a prevalência de obesidade grau 1 foi maior entre homens da área urbana. Estes achados são consistentes na literatura, visto que estas áreas diferem de acordo com suas características demográficas, culturais e socioeconômicas, o que determina a saúde dos sujeitos de diferentes maneiras^17^ ^,^ ^18^. Uma possível explicação para este achado se dá pela maior disponibilidade de alimentos ultraprocessados, poluição, privação de sono e estresse em áreas urbanas, com todos estes fatores fortemente associados ao excesso do acúmulo de gordura corporal^19^ ^,^ ^20^.

Em nossos achados, as mulheres de raça/cor negra no maior quartil de renda tiveram maior prevalência de obesidade quando comparadas a mulheres brancas no maior quartil de renda. Esse achado se soma a um corpo de evidências paradoxal, demonstrando maior prevalência de obesidade entre populações socialmente favorecidas em países de baixa e média renda^21^ ^,^ ^22^. Para mulheres negras de alta renda e escolaridade, a elevada prevalência de excesso de peso pode ser justificada por uma forma “oculta” de iniquidade, proveniente de uma estrutura racista, em que a discriminação pode gerar consequências negativas para o peso corporal e a saúde, diferente de seus pares^23^. Outra hipótese para o fato supracitado se dá pela possibilidade de mulheres pretas possuírem maior massa corporal e serem menos suscetíveis ao ideal de magreza branco ocidental. Desta maneira, mulheres pretas podem ser menos propensas a se envolverem em atividades para a modificação corporal que culminem na perda de peso^24^.

A epidemia de obesidade é multifatorial e seus determinantes são complexos^25^ ^,^ ^26^.

Vários fatores biológicos têm sido escrutinados, e muitos têm algum papel no acúmulo de gordura corporal, mas não há uma teoria que de fato possa explicar a epidemia. Um consenso vem predominando, que diz respeito ao conceito de balanço metabólico. Entretanto, dados populacionais (2000-2018) refutam a hipótese do balanço energético positivo, mostrando o aumento da obesidade de 31 para 42%, sem evidências de maior consumo de energia ou menor atividade física^27^ ^,^ ^28^.

Entre os vários fatores biológicos e teorias sobre o acúmulo de gordura, estão incluídas as exposições a mais de 50 obesógenos - produtos químicos que alteram a sinalização endócrina, os chamados disruptores, ou que alteram as funções do organismo, incluindo os níveis de insulina e a taxa metabólica - cujas alterações podem, inclusive, se propagar por gerações^29^. Há também farta documentação de que o consumo excessivo de açúcares e de produtos ultraprocessados leva ao ganho excessivo de peso^28^. Por outro lado, novos medicamentos que reduzem o apetite são promissores no que diz respeito a perda de peso e manutenção do peso perdido^30^.

Políticas, programas e diretrizes para a prevenção e controle da obesidade no Brasil, ainda que considerem sua multifatorialidade, possuem foco excessivo no aspecto biológico^31^. Tal fato ignora as intersecções de gênero/sexo, raça/cor, espaço geográfico e classe social, que contribuem sobremaneira para a predisposição ou agravamento da obesidade. O presente estudo lança luz sobre estas categorias pouco analisadas no que diz respeito a obesidade. Assim, indicamos quais fatores podem estar mais relacionados ao ganho de peso, o que podem colaborar no direcionamento de políticas de prevenção e controle da obesidade, como a Política Nacional de Alimentação e Nutrição^32^. Assim, as estratégias de vigilância nutricional, o monitoramento do risco de insegurança alimentar e a promoção da alimentação adequada e saudável se tornam mais direcionadas aos grupos desfavorecidos e podem tornar a Política Nacional de Alimentação e Nutrição mais eficaz e justa.

Apesar dos importantes achados, as limitações deste estudo incluem:

• a complexidade da autodeclaração da cor da pele/raça que pode não captar adequadamente o fenômeno racial;
• os dados são baseados na pesquisa de orçamentos familiares, que tem como principal objetivo investigar rendimentos e despesas entre famílias brasileiras, não possuindo variáveis específicas com foco em iniquidades de saúde;
• a informação sobre peso e estatura ter sido autodeclarada, portanto o viés de memória pode comprometer a acurácia dos dados.

Embora o peso e a altura sejam autodeclarados, ambos podem ser utilizados para calcular o IMC e classificar o estado nutricional de maneira válida e rápida em inquéritos populacionais^33^ ^,^ ^34^. Como pontos fortes, ressaltamos a representatividade populacional da Pesquisa de Orçamentos Familiares com dados demográficos e socioeconômicos que possibilitam avaliação das condições de saúde da população. Como se observa no presente estudo, ainda que a maior renda esteja historicamente associada ao aumento da oportunidade de recursos, a renda elevada pode influenciar a condição de saúde e nutrição de forma diferente a depender da raça e do gênero. Dessa forma, o presente trabalho soma ao campo da epidemiologia da obesidade por lançar luz a iniquidades, sobretudo, de acordo com raça/cor, gênero e renda.

Em conclusão, não encontramos diferenças entre os grupos situados em extremos de possível iniquidade no que diz respeito à obesidade. No entanto, a prevalência de obesidade grau I nos homens foi diferente de acordo com a área, sendo maior nos homens residentes da área urbana. Observou-se, ainda, uma maior prevalência de obesidade segundo a raça/cor, entre as mulheres pretas no maior quartil de renda. Entretanto, encontramos diferenças no padrão social de prevalência de sobrepeso e obesidade na população no que diz respeito ao sexo, raça/cor e renda.
